# *CD209* and Not *CD28* or *STAT6* Polymorphism Mediates Clinical Malaria and Parasitemia among Children from Nigeria

**DOI:** 10.3390/microorganisms8020158

**Published:** 2020-01-23

**Authors:** Olanrewaju B. Morenikeji, Jessica L. Metelski, Megan E. Hawkes, Anna L. Capria, Brooke N. Seamans, Catherine O. Falade, Olusola Ojurongbe, Bolaji N. Thomas

**Affiliations:** 1 Department of Biomedical Sciences, College of Health Sciences and Technology, Rochester Institute of Technology, Rochester, NY 14623, USA; 2 Department of Pharmacology & Therapeutics, College of Medicine, University of Ibadan, P.M.B 3017, Ibadan, Nigeria; 3 Department of Medical Microbiology and Parasitology, Ladoke Akintola University of Technology, P.M.B. 4000, Osogbo, Nigeria

**Keywords:** malaria, Africa, *CD209*, *CD28*, *STAT6*, children, host genetics, polymorphism, parasitemia

## Abstract

Malaria remains a significant disease, causing epic health problems and challenges all over the world, especially in sub-Saharan Africa. *CD209* and *CD28* genes act as co-stimulators and regulators of the immune system, while the *STAT6* gene has been reported to mediate cytokine-induced responses. Single nucleotide polymorphisms of these genes might lead to differential disease susceptibility among populations at risk for malaria, due to alterations in the immune response. We aim to identify key drivers of the immune response to malaria infection among the three SNPs: *CD209* (*rs4804803*), *CD28* (*rs35593994*) and *STAT6* (*rs3024974*). After approval and informed consent, we genotyped blood samples from a total of 531 children recruited from Nigeria using the Taqman SNP genotyping assay and performed comparative analysis of clinical covariates among malaria-infected children. Our results reveal the *CD209* (*rs4804803*) polymorphism as a susceptibility factor for malaria infection, significantly increasing the risk of disease among children, but not *CD28* (*rs35593994*) or *STAT6* (*rs3024974*) polymorphisms. Specifically, individuals with the homozygous mutant allele (*rs4804803G/G*) for the *CD209* gene have a significantly greater susceptibility to malaria, and presented with higher mean parasitemia. This observation may be due to a defective antigen presentation and priming, leading to an ineffective downstream adaptive immune response needed to combat infection, as well as the resultant higher parasitemia and disease manifestation. We conclude that the *CD209* gene is a critical driver of the immune response during malaria infection, and can serve as a predictor of disease susceptibility or a biomarker for disease diagnosis.

## 1. Introduction

Malaria remains a parasitic disease with about 3.2 billion people at risk of infection [1,2], with an estimated annual death toll of ~400,000 people out of 219 million clinical cases of malaria [3]. Many of these deaths occur among young children and pregnant women from sub-Saharan Africa [4,5]. Such mortality rates among these vulnerable groups necessitates timely intervention and improved control initiatives [6]. Several factors have been adduced for the significant morbidity and mortality rates [7], including ethnic delineation [8,9], parasite species/genotype [10,11,12,13,14], pathogen load [15], but most importantly, host genetic factors, which have been shown to mediate disease susceptibility, severity, and clinical outcome [16,17,18]. More than 90% of malaria infection cases in Africa are caused by *Plasmodium falciparum*, with multiple studies implicating the asexual blood stages of the parasite for its infection pathology [19,20]. The immune response against *P. falciparum*, in context of the host’s genetic contribution to disease outcome, is a complex process, and requires stepwise dissection.

To establish a protective immune mechanism against infection, there is the need for an intricately organized, innate and adaptive response, involving dendritic cells, macrophages, and B and T lymphocytes [4,21,22]. The *CD209* gene, also known as the dendritic cell-specific intercellular adhesion molecule-3-grabbing non-integrin (DC-SIGN) gene, encodes a transmembrane receptor on dendritic cells, and is a significant player in recognizing and presenting pathogens with a diverse evolutionary origin [23,24]. The CD209 protein has been shown to recognize pathogens through its N-terminal domain, binding to ligands on microbes in the process [25], as well as activating the signal transduction pathway in the process. Since DC’s are an essential component for antigen presentation, and for initiation of the process mediating adaptive immune responses, its polymorphisms, especially the *rs4804803* (*snp -336G/A*) gene promoter polymorphism, have been shown to modulate disease susceptibility or severity [26,27,28,29,30,31,32]. We have shown previously that this polymorphism tracks ethnicity, with significant genotypic and allelic diversity between African and American sickle-cell disease patients [27]. Importantly, this study provides one explanatory mechanism for the epidemiologic evidence of sickle-cell disease as a protective factor against malarial infection. We demonstrated that the preponderance of the homozygous recessive variant among Africans reveal a possible impairment of the immune response to infectious diseases (due to poor antigen presentation), and that this diversity is a critical factor determining susceptibility to disease or severity within and between groups.

*CD28*, on the other hand, is an important costimulatory molecule, promoting transcription signaling, activation and differentiation of naive CD4 and CD8 T cells, as well as activation of cytokines or cytokine receptors [33,34]. Studies have shown the significant co-stimulation of *CD28* in protective immune responses against various diseases [35,36,37,38], including malaria, with its polymorphisms also showing interethnic delineation mediating disease outcome. Though many of the single nucleotide polymorphisms have been identified and studied, only a few have been studied in the context of malaria infection [34,39]. Therefore, CD28 genetic polymorphisms may also serve an important role in the varying susceptibility to malaria.

Furthermore, published reports have shown that IL-10 production in response to a pathogenic stimulus is modulated by signal transducer and activator of transcription 6 (*STAT6*), enhancing IL-12 production in dendritic cells and driving a Th1 immune response in *STAT6*-/- mice [40,41]. The role of *STAT6* in the immune response to malaria infection is currently limited to a handful of studies, with no definitive conclusion [41,42]. A study examining regulatory gene variants in Congo showed a disparate response with *STAT6* and *FOXP3* among malaria-infected children [41], with the *STAT6* promoter variant *rs3024944* being associated with uncomplicated malaria, and the *FOXP3* SNP *rs11091253* being associated with parasitemia. The role of these regulatory variants in association with disease or covariates of infection has been very challenging to elucidate.

We have previously demonstrated the importance of the *CD14* promoter gene polymorphism (*rs2569190C/T*) [17] in malaria infection, showing that clinical malaria among Nigerian children is significantly regulated by this gene, in addition to its correlation with parasitemia, and thus a marker of disease severity. However, *CD14* gene polymorphisms are only one component of the complex host–parasite interaction, and thus constitute only a piece of the genetic puzzle required for a complete picture of the immune response to malaria. Considering the paucity of available data, what are the roles of *CD209*, *CD28* and *STAT6* gene polymorphisms, either individually or in combination, with malaria infection among children, and how are they associated with markers of disease severity (age, anemia and parasitemia)? Do these polymorphisms serve as susceptibility factors for malaria infection, or do they play any critical role in regulating disease covariates such as anemia and parasitemia in West Africa? To answer these questions, we evaluated the genetic variability of *CD209* (*rs4804803*), *CD28* (*rs35593994*) and *STAT6* (*rs3024974*) single nucleotide polymorphisms among malaria-infected children in Nigeria and extrapolated any association with markers of infection. Our data suggest that *CD209* (*rs4804803*) is a susceptibility factor for clinical malaria that also influences disease pathogenesis, but not *CD28* (*rs35593994*) or *STAT6* (*rs3024974*). We also show that despite the preponderance of *CD28* (*rs35593994*) wild type variants that would assist in T cell differentiation and parasite clearance, this was not the case. We postulate that a defective antigen presentation, depicted by the higher frequency of *CD209* mutant alleles, renders downstream immune response and parasite clearance moot. It also appears that any role for *STAT6* (*rs3024974*) variants to influence malaria pathogenesis occurs via a completely different mechanism, and this polymorphism is potentially still in flux.

## 2. Materials and Methods

### 2.1. Study Population and Genomic DNA Isolation

This study population consisted of febrile children recruited at St Mary Catholic Hospital, Ibadan, and in a rural primary health care center (Idi-Ayunre, Oluyole Local Government Area, Oyo State), southwest Nigeria, from November 2013 to November 2014, and in accordance with the 1975 Helsinki declaration. Detailed demographic, geographic and clinical parameters, as well as the genomic DNA extraction process, including modifications, are as described in (Ojurongbe et al., 2017). Approval was obtained from the University of Ibadan/University College Hospital Institutional Review Committee (approval number UI/EC/12/0279). Two hundred and thirty-one children presenting with malaria infection, for whom informed consent was obtained, were included into the study. Additionally, 330 matched uninfected children were recruited as controls, as part of the original study. Genomic DNA quality was accessed with a Nanodrop 2000 (ThermoFisher Scientific, Waltham, MA, USA). Axillary body temperature (in Celsius) and packed cell volume (expressed as a percentage), indicative of fever and anemia, respectively, were determined. Parasite count was evaluated per international standards [43].

### 2.2. TaqMan SNP Genotyping Assay

To perform the SNP genotyping analysis, our designed custom assay for *STAT6* (*rs3024974*; -*G* > *A*), alongside pre-made assays for *CD209* (*rs4804803*; -*383A* > *G*) and *CD28* (*rs35593994*; -*372G* > *A*) SNPs, were utilized. Briefly, a 10 µL reaction mixture was prepared for each assay containing 1 µL of the 20X Taqman SNP genotyping assay, 5 µL of 2× Taqman Mastermix (Thermo Fisher Scientific, Waltham, MA, USA), 1 µL of 20 ng genomic DNA and 3 µL of nuclease-free water. The reactions were set up for SNP genotyping using the CFXconnect (Bio-Rad, Hercules, CA, USA) real-time PCR machine with the following reaction conditions: 90 °C for 10 min, followed by 90 °C for 30 s, 56 °C for 30 s and 72 °C for 50 s, with a final extension of 72 °C for 5 min. Melt analysis was performed to confirm assay specificity; temperatures varied from 65 °C to 95 °C with increments of 0.5 °C for 5 s. We generated allele calls with the CFX Manager software.

### 2.3. Statistical Analysis

The program SNPstats [44] was used to estimate allelic and genotypic frequencies, testing the Hardy–Weinberg equilibrium for the three loci under study: *CD28* (*rs3559399*), *CD209* (*rs4804803*) and *STAT6* (*rs3024974*), with SNP’s rejected on the threshold of *p* < 0.05, as described by [45]; association analysis was performed for each SNP separately. Allelic and genotypic frequencies between controls and malaria-infected individuals were as previously described. To examine the association between malaria and the genetic variants of the loci under study with malaria, we utilized a binary logistic regression, to evaluate the association between gene variants and age, fever, PCV and parasitemia. Likewise, haplotype analysis was performed for the three SNPs; individuals who were heterozygous at more than one locus were excluded from the analysis.

## 3. Results

We genotyped blood samples collected from a total of 561 individuals (231 malaria-infected patients and 330 uninfected, control individuals) for *STAT6* (*rs3024974*), *CD28* (*rs35593994*) and *CD209* (*rs4804803*) gene polymorphisms using Taqman SNP genotyping assays. We also performed association analyses of genetic variants with clinical variables among the malaria-infected group. The genotypic frequencies of *STAT6*, *CD28* and *CD209* gene promoter polymorphisms in malaria patients were compared to the control group with logistic regression analysis (Table 1, Table 2 and Table 3). Our results show significant differences in genotypic frequencies between malaria and control groups for *CD28* (*rs35593994*; *p* = 0.0001) and *CD209* (*rs4804803*; *p* = 0.0001) but not *STAT6* (*rs3024974*; *p* = 0.30) gene polymorphisms. Although there was no significant difference in genotypic frequency between groups for the *STAT6* (*rs3024974*) gene, we did however observe a higher frequency of wild type *G*/*G* (74.4%) and heterozygote *G*/*A* (20.1%) variants in the control group, while the opposite (higher frequency of homozygous recessive variant *A*/*A*) was seen in the malaria group (8.7% versus 5.6% for the malaria and control groups, respectively (Table 1).

Examining genotypic frequency of the *CD28* (snp: *-372G/A; rs35593994*) gene polymorphism, we observed a higher frequency of homozygous mutant variants (*-372A/A*), though statistically insignificant in the control group compared to the malaria-infected group (16.8% versus 12.4% for the control and malaria groups, respectively) (Table 2); however, this difference was statistically insignificant. On the other hand, significantly more heterozygotes (*-372G/A*) were present in the malaria group (65.5%) compared to the control group (28.2%). Similarly, significantly more mutant variants (*-383G/G*) were observed in the malaria group (46.0%) compared to the control group (22.7%) for the *CD209* (snp: *-383A/G; rs4804803*) gene, depicting the homozygous mutant variant as a susceptibility factor for clinical malaria among children in southwestern Nigeria. This observation was not seen for other genes, except for *CD28* gene heterozygous variants (*372G/A*). The opposite was the case among those with the *CD209* gene homozygous dominant variant (*-383A/A*) (20.7% versus 52.1% for the malaria and control groups, respectively) (Table 3).

Our inheritance test model revealed that the *STAT6* (*rs3024974*) gene polymorphism did not increase the risk of malaria with the dominant (*G/A-A/A* versus *G/G*; OR = 0.98; 95% CI = 0.67–1.44) or recessive allele (*G/G-G/A* versus *A/A*; OR = 0.62; 95% CI = 0.32–1.20). However, in the case of the *CD28* (*rs35593994*) gene polymorphism, the dominant allele (*G/A-A/A* versus *G/G*; OR = 0.23; 95% CI = 0.16–0.34) demonstrated a significant increase in risk of malaria infection (*p* = 0.0001) but not the recessive allele (*G/G-G/A* versus *A/A*; OR = 1.43; 95% CI = 0.87–2.34) (Table 2). In the case of the *CD209* (*rs4804803*) gene polymorphism, a significantly increased risk of malaria infection was observed with both dominant and recessive alleles (*p* = 0.0001; Table 3).

To delineate the haplotype combination of the loci under study with the highest risk for malaria, we constructed a haplotype table, producing a total of eight haplotype combinations common to both the disease and control groups (Table 4). From our analysis, four out of the eight haplotype groups (haplotypes H2: *GGA*; H3: *GAG*; H4: *GGG*; and H8: *AGG*) showed a significant difference in haplotype frequencies between groups; others were insignificant. Based on the odds ratio, the same haplotypes (H2, H3, H4 and H8) in addition to haplotype H5 (*AAA; p* = 0.038) showed the highest/increased risk of malaria compared to others (Table 4), though the risk of malaria infection is significantly less with haplotype H8 (OR 0.12; 95% CI: 0.04–0.35). The linkage disequilibrium (LD) analysis showed the strongest association between *CD28* (*rs35593994*) and *CD209* (*rs4804803*) gene polymorphisms in the malaria group (0.77; *p* = 0.0022; Table 5).

**Figure 1:**
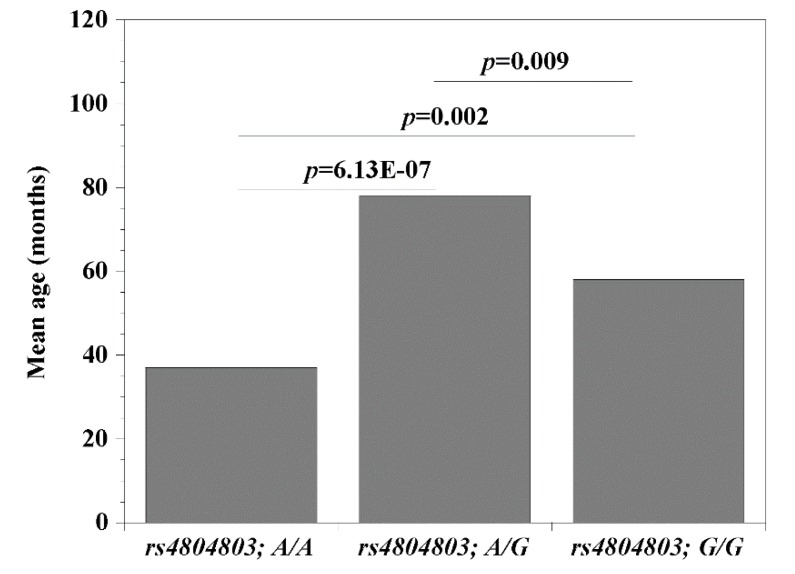
Mean age among malaria patients with *CD209 (*snp*: -383A* > *G; rs4804803*) wild type, heterozygous and mutant variants. Odds ratios were calculated by Fisher’s two-tailed exact tests. A *p*-value < 0.05 was considered significant.

**Figure 2:**
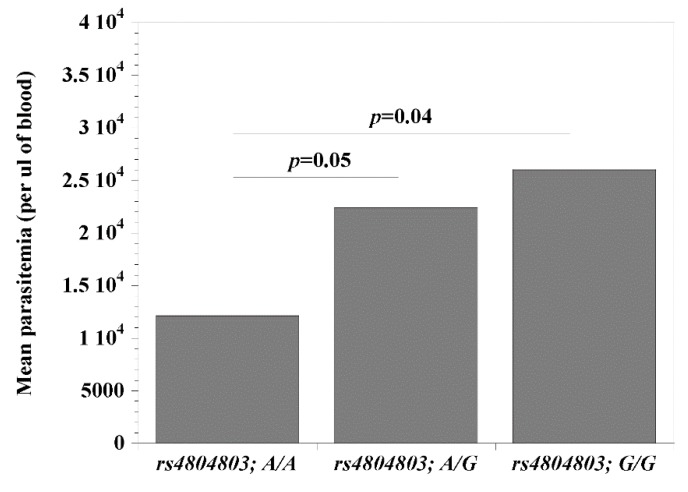
Mean parasitemia among malaria patients with *CD209* (snp: *-383A* > *G; rs4804803*) wild type, heterozygous and mutant variants. Odds ratios were calculated by Fisher’s two-tailed exact tests. A *p*-value < 0.05 was considered significant.

Since the *CD209* (*rs4804803*) gene polymorphisms presented the strongest association with malaria, we set out to examine the possibility that its genetic variants may be modulating the clinical covariates associated with disease (age, temperature (indicative of fever), packed cell volume, PCV (indicative of anemia) and parasitemia). Comparing wild type versus homozygous mutant variants, we report significant differences (*rs4804803A/A* < *rs4804803GG*) with age (*p* = 0.002; 37 versus 58 months; Figure 1) and parasitemia (*p* = 0.04; 12,139 versus 26,028 parasites per μL of blood; Figure 2). Malaria patients with the *CD209* gene wild type variants were much younger and had a lower mean parasitemia compared to patients with the mutant variant (older in age and a higher mean parasitemia). A similar observation with age and parasitemia, in addition to PCV (*p* = 0.005) was made when we compared patients with wild type variants (*rs4804803A/A)* to those with heterozygous (*rs4804803A/G*) variants (Table 6). To our surprise, the *STAT6* (*rs3024974*) gene showed a significant correlation between age and all its variants, and to some degree, with temperature, PCV and parasitemia (Table 6). The heterozygous variant of the *STAT6* gene promoter (*rs3024974G/A*) presented with the most protection against malaria infection (lowest mean age, PCV and parasitemia), possibly implying that the evolution of this gene in the context of malaria infection is still ongoing.

## 4. Discussion

Malaria is a life-threatening disease, causing global morbidity and mortality among endemic populations and non-immune individuals travelling to such locations, with the most deaths among children under five years of age [3,4,46]. Reports have shown that infection among children could lead to severe clinical episodes, therefore a better understanding of immunoregulation and/or the development of immunity post-infection is imperative. This is especially imperative in sub-Saharan Africa, where *Plasmodium falciparum*, the most virulent of the species, is endemic. Protective immunity can be acquired naturally after consecutive malaria infections when dendritic cells, serving as antigen-presenting cells, prime and activate the T cells necessary for downstream adaptive response, for which *CD209* and *CD28* molecules are significant participants [47,48,49]. Likewise, the *STAT6* gene is known to regulate the expression of regulatory T cells [50], while potentially modulating the immune response to malaria [41,42]. Therefore, elucidating the mechanism mediating susceptibility to or resistance against malaria would be significant in combatting disease pathogenesis. Studies on gene polymorphisms and genetic linkage analysis have shown differing relationships between genes or gene variants and association with disease susceptibility [41,51]. To this end, we investigated the role of *STAT6* (*rs3024974*), *CD28* (*rs35593994*) and *CD209* (*rs4804803*) gene polymorphisms in modulating disease susceptibility and infection outcome, as well as their relationship to clinical covariates of disease among children diagnosed with malaria in southwestern Nigeria. Remarkably, we show that the *CD209* (*rs4804803*) gene polymorphism is a susceptibility factor for malaria among the three genes under study, showing a significant disease-related association. The majority of malaria-infected children had the homozygous mutant variant (*rs4804803G/G*), while the control (uninfected) individuals predominantly had the wild type phenotype (*rs4804803A/A*), confirming our hypothesis that the *CD209* gene is a susceptibility factor for clinical malaria.

*CD209* encodes dendritic cell-specific ICAM3-grabbing non-integrin (DC-SIGN), and has been shown to bind various ligands including T-lymphocytes, thus contributing to activation of signal transduction pathways and recognition of infectious agents. Several studies have shown its polymorphic variants to be associated with tolerance or susceptibility to diseases, such as tuberculosis, schistosomiasis, leishmaniasis, and sickle-cell disease [23,27,30,51,52,53], asserting that the G allele significantly increased the risk of disease. Our results confirm this observation, indicating that mutation of the *CD209* gene has a considerable impact on malaria pathogenesis. Under normal circumstances, this gene protects against infection, but among the significant number of individuals with the mutant variant, this protection is lost, rendering these individuals more susceptible to disease. We show that a defective *CD209* response (mediated by G mutant allele) presented with significantly higher parasitemia and anemia (indicated by a low PCV) compared to the wild type variant, demonstrating its role in driving immune responses to malaria, more so than *STAT6* (*rs3024974*) or *CD28* (*rs35593994*) polymorphisms. This observation complements our previous report showing that the preponderance of the G mutant allele leads to a defective antigen presentation, weakening protective immune responses [27] and, as such, an unhindered parasitemia increase. On the other hand, the preponderance of the wild type A allele strengthens antigen presentation, T cell priming and downstream T cell differentiation, leading to an adaptive immune response that modulates disease (i.e., properly differentiated T cells drive a Th1 response that controls disease and parasitemia).

Though we found a notable diversity in *CD28* gene polymorphism, whereby the heterozygous variant was most common among malaria patients, and the homozygous dominant was most common among controls, this does not appear to be a factor in malaria pathogenesis. This observation implies a balanced immune response, expected to elicit a proper T cell response and control of parasitemia. The fact that this is not the case confirms our suspicion that higher parasitemia among malaria patients is hinged on the preponderance of the *CD209* mutant G allele, mediating a poor antigen presentation and an unmitigated disaster in the immune response. For the *STAT6* (*rs3024974*) gene, our analysis did not show any significant diversity in the genotypic frequency of the homozygous dominant (*G*/*G*), heterozygote (*G*/*A*) and the homozygous recessive (*A*/*A*) variants between the malaria and control groups (*rs3024974G/G* > *rs3024974G/A* > *rs3024974A/A*), except when analyzed in the context of disease covariates. The odds ratio showed no indication of an increased risk of malaria infection with the *STAT6* (*rs3024974*) gene polymorphism. This observation, however, contradicts previous studies associating this polymorphism with malaria pathogenesis among Congolese children [41], as well as cerebral malaria among children from Ghana [42]. As shown from our findings, the *STAT6* (*rs3024974*) polymorphism did not have any effect on disease susceptibility. Rather than driving a severe disease outcome as reported from Ghana, we propose a heterozygous advantage among malaria patients when analyzed with disease covariates. Patients with this variant, despite being the youngest (mean age: 46 months) and therefore expected to show the most severe disease outcome, had the lowest mean parasite count (8891 parasites per μL of blood compared to 24,952 and 22,523 parasites per μL of blood for homozygous dominant and homozygous recessive, respectively). This observation demonstrates the significantly higher relative fitness in endemic communities, endowed by the heterozygote genotype, but not seen in dominant or recessive groups, similar to that conferred by the sickle cell trait [27,54].

On the other hand, we report a low genotypic frequency of the *CD28* (*rs35593994*) homozygote recessive (*A*/*A*) variant in the malaria group, though statistically insignificant, implying that this variant possibly has a protective effect against infection. A published report has shown that low level parasitemia is retained in *CD28* knockout mice infected with malaria [55], suggesting that parasite control occurs via additional immune processes. It was reported that T cell memory is independent of *CD28,* and that *CD28*-deficient mice showed efficient type 1 and type 2 responses during infection with *Leishmania major* and *Heligmosoides polygyrus*, respectively [56,57]. The higher frequency of the heterozygote *G*/*A* variant in the malaria group might imply a potential additive effect producing a negative interaction of allele *G* and *A*, leading to a higher risk of disease in infected but not control groups. Therefore, the heterozygote variant in this case may have a negative effect on the immune response to disease and contribute to susceptibility, while the recessive variant (*A*/*A)* seems protective. Teutsch et al. (2004) described the *CD28* (*rs35593994*) SNP through identification of transcription factor binding sites, showing that the *A* allele possesses a CCAAT enhancer-binding protein site, which is lacking in allele *G.* Additional reports showed that this variant influences gene transcription and immune alteration in breast cancer [58,59]. We recommend that this polymorphism be further elucidated in the context of malaria and associated covariates of disease.

Interestingly, our linkage disequilibrium analysis revealed that *CD209* (*rs4804803*) and *CD28* (*rs35593994*) polymorphisms are significantly linked, justifying their co-stimulatory activity with pro-inflammatory cytokines such as *IL-4*, *IL-10*, *IL17F* and *TNF-α*. In the same vein, the haplotype combinations that identified haplotypes H2 (*GGA*), H3 (*GAG*) and H5 (*AAA*) as important for malaria might be as a result of the additive effect of the three loci under study favoring disease susceptibility. Importantly, the mutant allele *G* is significantly implicated among the haplotype definitions, implying that a change from an *A* to *G* as in the *CD209* (*rs4804803*) gene polymorphism will hamper downstream expression of the gene, thereby affecting the immune response and contributing to malaria pathogenesis, which was not seen in the *STAT6* (*rs3024974*) polymorphism. However, it is not impossible that *STAT6* (*rs3024974*) and *CD28* (*rs35593994*) polymorphisms may mediate malaria pathogenesis through a different mechanism not examined in this study. Overall, the *CD209* (*rs4804803*) gene polymorphism presenting the mutant allele *G* is critical in determining disease susceptibility, thereby identifying this gene and its polymorphism (*A* > *G*; *rs4804803*) as an important driver of immune response during infection.

## Figures and Tables

**Table 1:** Genotypic frequency of the *STAT6* gene promoter polymorphism between malaria-infected and control groups.

Polymorphism	Genotype	Malaria (*n* = 231)	Controls (*n* = 330)	Odds Ratio (95% CI)	*p*-Value
*STAT6* (*rs3024974*)	G/G	171 (74.0)	241 (74.4)	1	-
	G/A	40 (17.3)	65 (20.1)	1.15 (0.74–1.79)	0.3
	A/A	20 (8.7)	18 (5.6)	0.64 (0.33–1.24)	-
	**Dominant**				
	G/G	171 (74.0)	241 (74.4)	1	-
	G/A-A/A	60 (26.0)	83 (25.6)	0.98 (0.67–1.44)	0.92
	**Recessive**				
	G/G-G/A	211 (91.3)	306 (94.4)	1	-
	A/A	20 (8.7)	18 (5.6)	0.62 (0.32–1.20)	0.16
	**Overdominant**				
	G/G-A/A	191 (82.7)	259 (79.9)	1	-
	G/A	40 (17.3)	65 (20.1)	1.20 (0.77–1.85)	0.16

**Table 2:** Genotypic frequency of the *CD28* gene promoter polymorphism between malaria-infected and control groups.

Polymorphism	Genotype	Malaria (*n* = 231)	Controls (*n* = 330)	Odds Ratio (95% CI)	* p*-Value
*CD28* (*rs35593994*)	G/G	50 (22.1)	173 (54.9)	1	-
	G/A	148 (65.5)	89 (28.2)	0.17 (0.12–0.26)	0.0001
	A/A	28 (12.4)	53 (16.8)	0.55 (0.31–0.95)	-
	**Dominant**				
	G/G	50 (21.1)	173 (54.9)	1	-
	G/A-A/A	176 (77.9)	142 (45.1)	0.23 (0.16–0.34)	0.0001
	**Recessive**				
	G/G-G/A	198 (87.6)	262 (83.2)	1	-
	A/A	28 (12.4)	53 (16.8)	1.43 (0.87–2.34)	0.15
	**Overdominant**				
	G/G-A/A	78 (34.5)	226 (71.8)	1	
	G/A	148 (65.5)	89 (28.2)	0.21 (0.14–0.30)	0.0001

**Table 3:** Genotypic frequency of the *CD209* gene promoter polymorphism between malaria-infected and control groups.

Polymorphism	Genotype	Malaria (*n* = 231)	Controls (*n* = 330)	Odds Ratio (95% CI)	*p*-Value
*CD209* (*rs4804803*)	A/A	44 (20.7)	163 (52.1)	1	-
	A/G	71 (33.3)	79 (25.2)	0.30 (0.19–0.48)	0.0001
	G/G	98 (46)	71 (22.7)	0.20 (0.12–0.31)	
	**Dominant**				
	A/A	44 (20.7)	163 (52.1)	1	-
	A/G-G/G	169 (79.3)	150 (47.9)	0.24 (0.16–0.36)	0.0001
	**Recessive**				
	A/A-A/G	115 (54)	242 (77.3)	1	-
	G/G	98 (46)	71 (22.7)	0.34 (0.24–0.50)	0.0001
	**Overdominant**				
	A/A-G/G	142 (66.7)	234 (74.8)	1	-
	A/G	71 (33.3)	79 (25.2)	0.68 (0.46–0.99)	0.044

**Table 4:** Estimated haplotype frequencies of the selected loci between the malaria-infected and control groups.

Haplotype	Haplotype Definition			Haplotype Frequency				
	* STAT6* (*-G* > *A*)	* CD209* (*-383A* > *G*)	* CD28* (*-372G* > *A*)	Malaria	Controls	Malaria vs. Controls	OR (95% CI)	* p*-Value
H1	G	A	A	0.1431	0.3688	0.2794	1	NA
H2	G	G	A	0.3053	0.2261	0.2556	0.41 (0.27–0.61)	0.0001
H3	G	A	G	0.1559	0.17	0.1612	0.43 (0.27–0.71)	0.001
H4	G	G	G	0.2225	0.0791	0.1408	0.20 (0.13–0.31)	0.0001
H5	A	A	A	0.0663	0.0643	0.0649	0.49 (0.25–0.96)	0.038
H6	A	A	G	0.008	0.0442	0.0293	2.09 (0.27–16.22)	0.48
H7	A	G	A	0.0341	0.0308	0.032	0.48 (0.21–1.11)	0.086
H8	A	G	G	0.0648	0.0166	0.0369	0.12 (0.04–0.35)	1 × 10^−4^

**Table 5:** Linkage disequilibrium analysis of *STAT6*, *CD209* and *CD28* genetic polymorphisms.

D-Stat/*p*-Values	*STAT6* (*rs3024974*)	*CD209 (rs4804803)*	*CD28* (*rs35593994*)
*STAT6* (*rs3024974*)	-	0.0075	0.0103
*CD209 (rs4804803)*	0.187	-	0.0022
*CD28* (*rs35593994*)	0.0582	0.7655	-

**Table 6:** Comparative analysis of disease covariates with genetic variants of selected loci among malaria-infected patients.

Polymorphism	Genotype	Age (Months)	Temperature (Celsius)	PCV (%)	Parasitemia (per μL of blood)
***STAT6*** (***rs3024974***)	GG	59	38	34	24,952
	GA	46	38	31	8891
	AA	96	38	34	22,523
	**Mean** **Total**	**201**	**114**	**99**	**56,366**
***p*** **-value**	GG vs. GA	**0**.**0552**	**0.0272**	**0.0539**	**0.0012**
	GG vs. AA	**0**.**0035**	0.1474	0.7942	0.7357
	GA vs. AA	**0.0003**	**0.0096**	**0.0083**	**0.0568**
***CD28*****(snp*****: -372G/**A*)	GG	77	38	34	14,956
	GA	53	38	34	24,279
	AA	67	38	32	19,727
	**Mean Total**	**197**	**114**	**100**	**58962**
***p*** **-value**	GG vs. GA	0.0708	0.4436	0.2428	0.4489
	GG vs. AA	0.3617	0.4131	0.1878	0.4521
	GA vs. AA	0.0211	0.1292	0.7745	0.1315
***CD209*****(snp*****: -383A/G***)	AA	37	38	30	12,139
	AG	78	38	33	22,415
	GG	58	38	36	26,028
	**Mean Total**	**173**	**114**	**99**	
***p*** **-value**	AA vs. AG	**6.1306 ×** **10** ^**−4**^	0.3679	**0.0045**	**0.0495**
	AA vs. GG	**0.0019**	0.2925	0.0941	**0.0399**
	AG vs. GG	**0.0094**	0.8312	0.4369	0.5983
